# Real-Time Therapy Response Monitoring Using Surface Biomarkers on Circulating Tumor Cells

**DOI:** 10.3390/cancers18030391

**Published:** 2026-01-27

**Authors:** Saloni Andhari, Jaspreet Farmaha, Ashutosh Vashisht, Vishakha Vashisht, Jana Woodall, Ashis K. Mondal, Kimya Jones, Ajay Pandita, Gowhar Shafi, Mohan Uttarwar, Jayant Khandare, Ravindra Kolhe

**Affiliations:** 1 Department of Pathology, Medical College of Georgia, Augusta University, Augusta, GA 30912, USA; sandhari@augusta.edu (S.A.);; 2 1Cell.Ai, Wakad, Pune 411057, India; 3 1Cell.Ai, 320 Hatch Drive, Foster City, CA 94404, USA; 4 Actorius Innovations and Research Co., 11842 Churchill Way, Porter Ranch, CA 91326, USA

**Keywords:** circulating tumor cells, surface biomarkers, circulating tumor DNA, minimal residual disease

## Abstract

Circulating tumor cells (CTCs) are shed from the primary tumor into the bloodstream and represent dynamic molecular biomarkers for monitoring the progression of cancer. While profiling tumor tissues with over expression of cell surface markers, such as PD-L1 or HER2, is standard in guiding therapy, tissue samples are often inaccessible and inadequate, especially post-surgery or in cases of recurrence. Emerging clinical evidence indicates that CTC counts and biomarker surface expression can predict prognosis and therapeutic resistance more accurately than imaging or tissue-based approaches. Recent advancements in the CTC detection methods, based on physical properties or surface markers (e.g., EpCAM), coupled with next-generation sequencing (NGS) have enabled the isolation of these rare cells and their molecular characterization. Consequently, CTCs provide a real-time alternative, enabling repeated, longitudinal assessment of tumor phenotype and therapeutic response. This review emphasizes the translational potential of surface protein biomarkers on CTCs for profiling, namely PD-L1, HER2, and EGFR, as a clinically actionable approach to stratify patients, guide immunotherapy decisions, and monitor minimal residual disease (MRD), especially when longitudinal tissue biopsies are not feasible.

## Simple Summary

Circulating tumor cells (CTCs), cancer cells shed from primary tumors into the bloodstream, are emerging as dynamic, non-invasive biomarkers for real-time cancer monitoring, especially when tissue biopsies are inaccessible or inadequate. Unlike static tissue samples, CTCs allow repeated assessments that track tumor evolution, therapeutic response, and minimal residual disease. Hence, CTCs offer a minimally invasive, real-time alternative to tissue biopsies for cancer monitoring, particularly through surface protein biomarkers like PD-L1, HER2, and EGFR. As detection technologies improve and the clinical relevance of CTC continues to be established, CTC profiling is poised to significantly influence the future of precision oncology.

## 1. Introduction

Over the past few decades, the detection and monitoring of minimal residual disease (MRD) has become a cornerstone in cancer surveillance. Despite curative intent, cancer recurrence remains a major threat due to undetected MRD. In 2021, an estimated 2.65 million new cases of early-onset cancer were diagnosed worldwide, except non-melanoma skin cancer (NMSC) [1]. These cases accounted for approximately 0.99 million deaths and 50.7 million disability-adjusted life years (DALYs). The greatest DALY burden was attributable to breast cancer, followed by cancers of trachea, bronchus and lung (TBL), cervix, colon, and stomach. The diverse array of analytes in blood can be investigated to monitor MRD, which include circulating tumor cells (CTCs), circulating tumor DNA (ctDNA), extracellular vesicles (EVs), and tumor educated platelets [2,3,4,5].

In 1829, Jean Claude Recaimer used the term metastasis to explain the spread of cancer from one organ to another [6], while in 1869, Thomas Ashworth was the first to observe cells resembling cancer cells in the blood sample of a patient with multiple subcutaneous tumors [7]. He concluded that if the cancer cells in the blood originated from a pre-existing tumor, they must have traveled through the entire circulatory system before reaching the internal saphenous vein where he collected the blood sample.

Among the liquid biopsy modalities, CTCs have emerged as a promising tool, offering presence-based detection and molecular phenotyping to guide treatment selection and response monitoring in real time [8]. Moreover, advances in combinatorial approaches of nanoscale imaging such as atomic force microscopy and microfluidics could potentially serve as an efficient prognosis approach to detect and isolate CTCs with intact phenotypes [9]. ctDNA and CTCs act as a dynamic duo for residual disease tracking and enable adjuvant chemotherapy decisions and early detection of recurrence. This is evidenced by robust clinical data from trials like DYNAMIC, BESPOKE, GALAXY, MIRACLE, and BRE12-158 [10,11,12,13,14,15,16].

Recently, an international panel of cancer specialists reached an agreement that testing for circulating tumor cells is clinically valuable for predicting outcomes and tracking therapeutic response in advanced breast and prostate cancers, though the technology needs further validation in other malignancies and earlier disease stages [17]. The experts identified several obstacles limiting broader adoption, including the need for better detection methods, uniform testing procedures, and stronger clinical data. Furthermore, they advocate for a shift toward analyzing the molecular characteristics of these cells rather than merely counting them and integrating CTCs with cell-free DNA for comprehensive liquid biopsy approaches. In line with these principles, platforms such as OncoMonitor (1Cell.Ai, Pune, India), integrate cellulomics and genomics improving sensitivity, risk stratification, and treatment guidance across cancers thus enable personalized surveillance [18].

Metastasis has been considered to involve a molecular cascade where cancer cells extravasate the primary tumor as CTCs and colonize distant organs by adapting to the local microenvironment to form a secondary lesion [19,20]. Emerging viewpoints conceptualize cancer as an ecosystem and metastasis as an ecological process spanning primary, circulatory, and other interacting ecosystems, moving beyond a purely molecular interpretation [21]. The proposed ecology theory and cancer ecology tree introduce a new framework for elucidating cancer’s complex causal pathways.

However, metastasis is yet to be completely understood and results in the death of more than 90% of patients [22,23]. A substantial number of cancer patients, despite being free of detectable metastasis, will relapse into metastatic disease within 5 years of tumor resection [24]. This indicates the presence of MRD in patients who appeared to have undergone successful initial first-line cancer therapies. For example, the risk of relapse in stage III breast cancer patients after 5 years of adjuvant therapy is 13% [25]. In addition, approximately 20–50% stage II-III colorectal cancer (CRC) patients develop recurrence and metastasis after comprehensive treatment [26,27].

**Figure 1:**
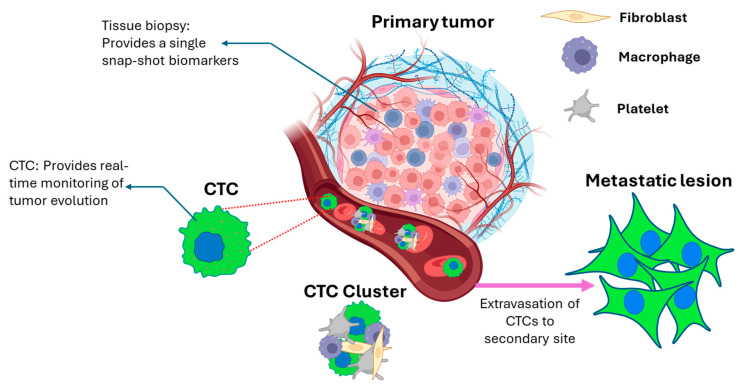
Schematic illustration of cancer metastasis from the primary tumor to form a metastatic lesion. CTCs escape the primary tumor, travel through the bloodstream, and reach distant organs to form secondary tumors. The CTCs in blood may be present as solitary/single cells or as clusters. The clusters have been shown to have greater metastatic potential compared to single CTCs. The primary tumor tissue biopsy and CTCs can be utilized to determine the characteristics of the cancer in different ways. While the access to primary tumor tissue is static, CTCs are dynamic in nature and can be analyzed throughout the treatment regimen. Created in BioRender. Andhari, S. (2026) https://BioRender.com/d45z0u2, accessed on 21 January 2026.

The role of CTCs in monitoring and prognosis of cancer is evolving rapidly, and understanding their phenotype and genotype is crucial. Singh et al. elaborated on various chemo-specific designs for enumerating CTCs and highlighted surface markers that can be used to target CTCs [28]. In contrast, Masmoudi et al. recently described the “surfaceome” of CTCs concerning their immune evasion potential [29]. While tissue-based molecular profiling remains a diagnostic mainstay, its static nature and procedural limitations restrict its use for serial monitoring. The CTC biomarker profiling addresses this gap, offering actionable insights into tumor evolution, therapy resistance, and metastatic potential with minimal invasiveness (Figure 1). Here, we discuss the origin of CTCs and describe different methods of detecting them. We highlight the importance of enumerating the CTCs in blood. Finally, we discuss the importance of analyzing established and emerging surface markers on CTCs and their role in disease monitoring.

## 2. Mechanism of Cancer Metastasis

Since its first description by Recaimer, the mechanism of metastasis has been extensively studied and is considered one of the hallmarks of cancer [30]. Over the years, multiple models have been proposed to describe the metastatic process, which is yet to be completely understood [31]. A series of sequential steps are considered to be involved in the spread of cancer from the primary tumor to a distant organ. A process known as the epithelial–mesenchymal transition (EMT) imparts migratory potential to epithelial cancer cells [32]. The epithelial cells lose their intercellular adhesion and cell–extracellular matrix adhesion properties due to EMT and invade the surrounding local tissue. The migration of cancer cells is promoted by factors such as oxygen deficiency and genetic mutations (mutant p53) [33,34,35]. The cancer cells stimulate angiogenesis through the secretion of chemokines and other pro-inflammatory molecules which draw immune cells to the tumor site [36]. The newly formed blood vessels are the primary route of dissemination of cancer cells into the blood circulation. The blood vessels are highly permeable (“leaky”) due to fragmented basement membranes and are hyperbranched, thereby aiding the intravasation of cancer cells from the primary tumor into the blood; these cancer cells in blood are referred to as CTCs [37]. It has been demonstrated that the cancer cells undergoing metastasis depict plasticity and are in a hybrid epithelial or mesenchymal state, capable of interconverting in either cell type [38]. The epithelial state enables intravasation; however, a reversal of EMT, mesenchymal-to-epithelial transition (MET), aids extravasation to establish distant metastatic lesions.

Metastasis has classically been associated with single-cell dissemination driven by EMT and followed by MET. Nonetheless, other dissemination strategies are increasingly recognized, including collective or cluster-based migration and invasion, in which cells can retain intercellular junctions and may not display a strong induction of mesenchymal markers, despite acquiring migratory and invasive capacities [39,40]. Moreover, the metastatic outgrowth of disseminated tumor cells can proceed through mechanisms that do not rely on MET [41]. Taken together, accumulating evidence points to a more complex and context-dependent role for EMT/MET in cancer metastasis. In certain settings, these programs are indispensable, whereas in others, they are not strictly required and instead act in permissive or potentially catalytic ways, shaping cellular phenotypes that accelerate tumor cell escape and successful colonization [42,43,44].

Whichever pathway the metastatic process may follow, it provides an opportunity to capture, isolate, and analyze CTCs during various stages of the disease. The enumeration and analysis of CTCs at the early stage may hint towards the progression of disease. On the other hand, the analysis post treatment can assist in altering treatment strategies based on any observed resistance markers. The strategies to detect CTCs are discussed in the next section.

## 3. CTCs and Methods of Detecting CTCs

CTCs are very rare cells, making it difficult to isolate them. The CTC count can be as low as one cell amongst billions of blood cells in 7.5 mL of blood [45]. Isolation technologies concentrate and extract CTCs from large numbers of blood cells, typically using gradient centrifugation or red-blood-cell lysis, then further enriching them based on physical traits or antibody-driven negative or positive selection [46].

### 3.1. Bio-Physical Approaches of CTC Isolation

CTCs’ morphology, such as their large size (10 µm to 30 µm) compared to normal blood cells, is often exploited to isolate them from a blood sample [47,48]. The CellSieve Microfilters (Creatv Bio, Division of Creatv Micro Tech Inc., Rockville, MD, USA) use porous membranes with a pore diameter of 7 µm to isolate CTCs with a capture efficiency of 90% when MCF-7 cells were spiked in blood [49]. Another membrane filter-based CTC isolation technology, ScreenCell (Screen Cell, Paris, France), showed a recovery rate of 55% and 100% specificity when breast cancer cells MDA-MB-231 were spiked in blood [50]. Parsortix technology (CelLBx Health, Guildford, UK) is a microfluidic cassette that captures CTCs based on their size. When MCF-7 cells were spiked in blood, the capture rate was 58% using the Parsortix microfluidic cassette [51]. Additionally, density gradient centrifugation can be used to isolate CTCs based on the difference in sedimentation rates of different cells in media of varying viscosities [52,53]. Density gradient-based separation as a standalone technique usually yields low recovery of CTCs and has been used with porous membranes OncoQuick (Greiner Bio-One North America Inc, Monroe, NC, USA), to improve the recovery rate.

Gascoyne et al. demonstrated that dielectrophoretic field flow fractionation (depFFF) can isolate rare, viable tumor cells from blood without antibodies or labels by exploiting differences in cell membrane capacitance between normal cells and cancer cells [54]. Using a scaled-up dielectrophoretic (DEP) chamber, the authors separated three breast cancer cell lines from mixtures containing millions of peripheral blood mononuclear cells. They demonstrated over 90% tumor cell recovery under low loading conditions and showed that the recovered cells remained viable and capable of regrowth.

The bio-physical methods of CTC isolation offer ease of processing by reducing sample preparation steps required for affinity-based approaches. However, the isolated CTCs may be contaminated with other blood cells, thus having reduced specificity.

### 3.2. Receptor–Ligand Interaction-Based Approaches

CellSearch (Menarini Silicon Biosystems, Bologna, Italy) is the first FDA-approved platform that utilizes anti-EpCAM antibody immunomagnetic beads to isolate and enumerate CTCs [55]. Early studies established the recovery of spiked cells at greater than 85% using CellSearch, which has since been used in multiple clinical trials to determine prognosis in various cancer types [56]. OncoDiscover (Actorius Innovations and Research, Pune, India) is an affinity-based magneto-graphene nanoplatform, approved by the Central Drug Standards Control Organization (CDSCO), India, which can be used as a medical device for CTC isolation and enumeration in clinical settings [57]. The platform utilizes anti-EpCAM antibody to capture cancer cells of epithelial origin. The recovery of spiked cells has been reported to be greater than 88%, and 84% of the cancer patients evaluated showed the presence of at least 1 CTC. Affinity-based approaches have the advantage of targeting CTCs with high expression of specific ligands, thus reducing the possibility of capturing non-specific blood cells. On the other hand, the use of antibodies may increase the overall cost of the assay.

While enrichment approaches based on EpCAM have laid the foundation for CTC research, they are limited in their ability to recover cells that have undergone EMT. This shortcoming has prompted the development of complementary strategies that better reflect the biological diversity of CTC populations. Expanding enrichment panels to include markers such as EGFR and vimentin has been shown to provide a broader view of CTC heterogeneity, with CTCs in EMT phase being captured more efficiently using vimentin antibody-based capture platforms [58,59]. In parallel, the marker-independent approaches described above avoid the biases inherent to marker-based selection and often maintain higher cell viability, which is advantageous for downstream applications including ex vivo culture and molecular analyses (Table 1). In contrast, immunoaffinity-based techniques can compromise cell integrity because of antibody interactions.

Selecting an appropriate CTC isolation strategy therefore depends on the intended research or clinical objective, taking into account factors such as the need to recover diverse CTC subpopulations, acceptable purity thresholds, and planned downstream assays. The use of negative selection methods, which remove unwanted blood cells, can further enhance enrichment by increasing the relative abundance of viable CTCs. Overall, these developments highlight the value of integrated, multimodal isolation approaches that account for the intrinsic heterogeneity of CTCs. The new-age CTCs isolation platforms must be envisioned to capture CTCs more consistently and preserve their integrity to aid downstream genomics, transcriptomics, and proteomic analysis.

## 4. Role of CTC Abundance in Prognosis of Carcinomas

Multiple clinical trials demonstrating the utility of CTC enumeration using CellSearch technology in the prognosis of various carcinomas led to its approval by the FDA. Cristofanilli et al. evaluated the hypothesis, whether the number of CTCs observed in metastatic breast cancer patients can predict survival [61]. One hundred and seventy-seven patients with metastatic breast cancer measurable via regular imaging technologies were recruited for this study. CTC counts at baseline and, at the first follow-up visit, were the strongest predictors of progression-free survival (PFS) and overall survival (OS), indicating that pre-treatment CTC counts independently forecast PFS and OS in metastatic breast cancer.

Cohen et al. recruited 430 patients with metastatic colorectal cancer (mCRC) and enumerated CTCs in the peripheral blood in a similar study [62]. Patients were classified with favorable or unfavorable prognosis based on three or more CTCs or less than three CTCs per 7.5 mL of blood. After adjustment for the clinically relevant variable, the baseline and follow-up CTC levels remained strong predictors of PFS and OS. Similarly, the CTC counts measured before and during therapy independently predicted PFS and OS in this patient cohort. Thus, the information on CTC abundance helped in predicting the progression of disease.

**Figure 2:**
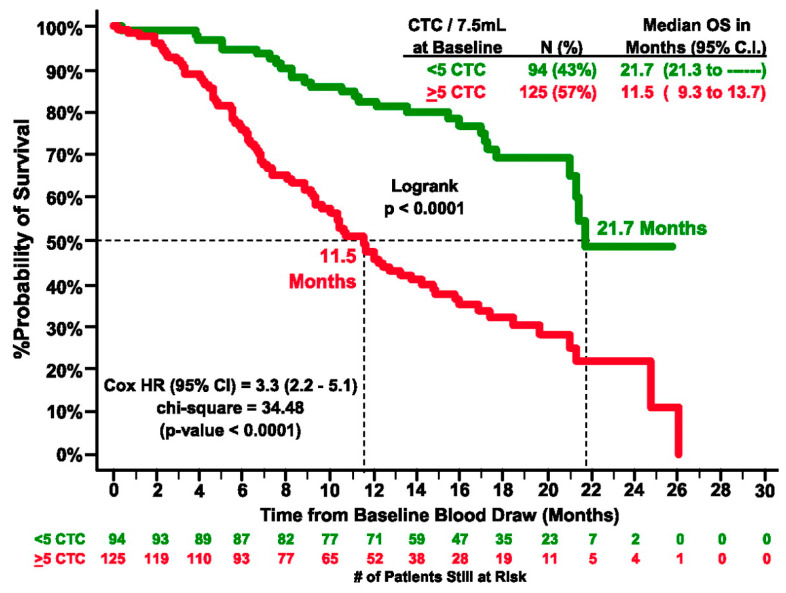
Kaplan–Meier estimates of probabilities of OS of castration-resistant prostate cancer patients. In the graph # represents number. (Reprinted from [63], with permission from AACR).

In yet another study, Bono et al. enrolled 276 patients with castration-resistant prostate cancer (CRPC). They stratified the patients into favorable or unfavorable groups based on the number of five or more or less than five CTCs per 7.5 mL of blood [63]. Patients with initially unfavorable CTC counts who shifted to favorable levels showed a marked improvement in prognosis (6.8 to 21.3 months). In contrast, those with favorable baseline counts that became unfavorable experienced a decline in outcomes (>26 to 9.3 months). Thus, CTCs were determined to be independent predictors of OS in CRPC patients (Figure 2).

CTC enumeration has also been reported in lymphatic fluid by Han et al., successfully identifying lymphatic CTCs mouse models of melanoma and breast cancer using highly sensitive in vivo photoacoustic and fluorescence-based flow cytometry [64]. They demonstrated that extremely small primary tumors were capable of releasing lymphatic CTCs, with the smallest measured tumor producing approximately one lymphatic CTC every 30 min. Thus, the presence of CTCs in lymphatic fluid and their enumeration along with CTCs in the blood compartment can be used for disease prognosis in the future, pending evaluation in larger cohorts.

## 5. Molecular Markers Associated with CTCs

While the potential of enumerating CTCs has been highlighted by multiple clinical studies in the previous sections, recent investigations reveal the molecular heterogeneity in CTC population [65]. Additionally, as discussed in Section 2, different mechanisms are involved in the metastatic journey of CTCs. From the time of dissemination in blood and until the formation of metastatic lesion at a distant site, CTCs undergo metabolic changes and some of the changes result in the upregulation or downregulation of certain surface biomarkers. EMT-related changes include the downregulation of EpCAM and upregulation of vimentin, CD44 [66]. CTCs achieve evasion of immune surveillance by driving the expression of molecules such as programmed cell death ligand 1 (PD-L1) [67]. These changes have been demonstrated to be cancer-specific. However, such changes can be exploited to predict the progression of disease and/or to provide targeted therapies.

Furthermore, a recent study demonstrated that, unlike primary tumor cells, CTCs differ in metabolic activity and show an anti-Warburg effect [68]. The authors performed a systematic bioinformatic analysis of publicly available bulk and single-cell RNA sequencing datasets from multiple cancers. The analyses revealed that CTCs from melanoma, prostate cancer, and lung adenocarcinoma showed significantly higher oxidative phosphorylation (OXPHOS) relative to glycolysis compared with primary tumors or metastases. Importantly, higher anti-Warburg effect scores in CTCs correlated with disease progression and poorer therapeutic response in melanoma and prostate cancer patients. These results suggest that the anti-Warburg effect may have functional and clinical relevance, potentially supporting CTC survival in circulation and enhancing metastatic capacity. It must be noted that further prospective validation studies across additional cancer types and datasets are needed to establish the significance of the anti-Warburg effect.

Thus, going forward, clinical tests which provide the molecular profiles of CTCs along with their abundance in blood will accurately depict the tumor landscape of a patient. In this section we discuss the various molecular markers associated with CTCs which may help in predicting the therapeutic outcome.

### 5.1. Programmed Cell Death Ligand 1 (PD-L1)

**Figure 3:**
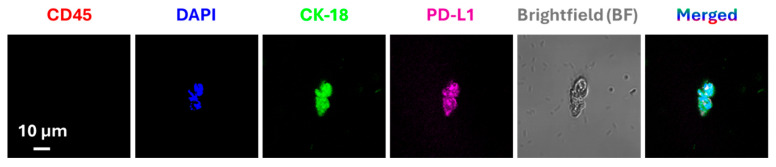
Immunofluorescence-based detection of CTCs and analysis of expression of PD-L1. Representative CTC image isolated from a lung cancer patient using an immunomagnetic platform. The captured CTCs were immuno-stained with anti-PD-L1 and anti-cytokeratin 18 (CK-18) antibodies. Cells with positive CK-18, DAPI fluorescence signals and negative CD45 fluorescence signal, were considered CTCs. Reproduced from [57].

**Figure 4:**
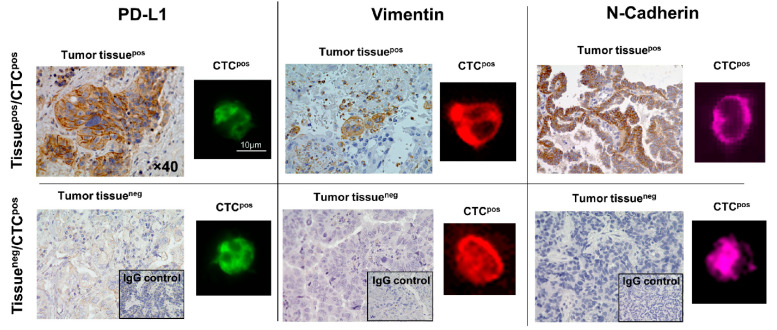
Immunofluorescence-based analysis of CTCs for the expression of PD-L1, Vimentin, and N-Cadherin compared with IHC staining of tissues obtained from patients with NSCLC. As observed in the lower panel, even when the tumor tissue is negative (tumor tissue^neg^) for surface biomarkers, CTCs are positive (CTC^pos^). This reinforces the importance of monitoring the disease using dynamic analytes such as CTCs. The IHC images were captured at 40× magnification. In the fluorescent images the scale bar represents 10 µm. In the fluorescent images, green represents PD-L1 positive CTCs, red represents vimentin positive CTCs and purple represents N-Cadherin positive CTCs. Reprinted from [88] (Open access under the Creative Commons CC BY license; https://creativecommons.org/licenses/by/4.0/, accessed on 21 January 2026).

The programmed cell death protein (PD-1) is a transmembrane protein found on the surface of activated T cells, B cells, monocytes, and other immune cells, while its ligand [69,70], programmed cell death ligand 1 (PD-L1), is expressed in heart tissues, lung tissues, and tumor cells [69,70,71,72]. Activation of PD-1 by PD-L1 leads to T-cell anergy, enabling immune escape of PD-L1-expressing tumor cells [72]. Remarkable success in anti-cancer therapy has been achieved in recent years by employing therapeutic antibodies targeting the PD-1/PD-L1 interaction, with the FDA approving numerous immune checkpoint inhibitors (ICIs). Antibodies targeting PD-1 include Nivolumab, Pembrolizumab, and Cemiplimab, and antibodies targeting PD-L1 include Atezolizumab, Avelumab, and Durvalumab [73,74,75,76,77]. However, a broad variation in response has been noted, with occasional failure of the anti-PD-1/PD-L1 therapy, which can be attributed to poor or aberrant expression of PD-L1. Studies reviewing the ICI therapy have revealed insightful traits such as increased PD-L1 expression in tumor cells following ICI therapy, which may contribute to acquired resistance to immunotherapy. Multiple groups have reported the presence of PD-L1 on CTCs isolated from patients with different types of cancers, namely, lung, breast, prostate, and colon (Figure 3 and Figure 4) [57,78,79,80]. Hence, real-time monitoring of PD-L1 expression on CTCs offers a valuable approach to analyze the temporal and dynamic changes in tumor surface markers in response to immunotherapy.

To generate clinical evidence supporting this approach, recent studies have compared the expression of PD-L1 in tissue biopsy to CTCs. Andhari et al. compared PD-L1 expression on CTCs in 30 NSCLC patients by immunofluorescence and on tissue biopsies by IHC [81]. CTCs were isolated from NSCLC patients using an anti-EpCAM antibody-based enrichment platform. A 50% concordance was observed between PD-L1 expression on CTCs and tissue specimens, with a significant heterogeneity of PD-L1 expression observed both within and between patients. Similarly, Guibert et al. used a size-based CTC isolation technique to capture and stain the CTCs from advanced NSCLC patients and compared the expression with tissue biopsies [82]. A significant discordance was observed between PD-L1 expression on CTCs and tissue biopsies, with a higher percentage of false negatives observed in the tissue biopsies. While simply having PD-L1-expressing CTCs at baseline did not show a significant association with clinical endpoints, patients who progressed within six months tended to have a greater proportion of PD-L1-positive CTCs at the outset (≥1%) (*p* = 0.04). Additionally, at the time of disease progression, every patient exhibited PD-L1-positive CTCs. These examples highlight the role of CTCs in guiding clinical decisions and using chemotherapeutics or immune checkpoint inhibitors.

The clinical relevance of PD-L1 expression on CTCs as a potential biomarker for predicting the interventional immunotherapy outcomes has also been recently reviewed across multiple cancer types [83]. Recently PD-L1 expression on CTCs and circulating white blood cells obtained from 106 NSCLC patients showed concordance with the PD-L1 presence in paired tumor tissue samples [84]. In another study, Koutsodontis et al. reported a quantitative real-time RT-qPCR assay for measuring the PD-L1 expression on CTCs isolated from 70 head and neck squamous cell carcinoma (HNSCC) patients, revealing a negative association between PD-L1 expression and progression-free survival (PFS) [85]. Vasudevan et al. studied PD-L1 expression on CTCs and CTC clusters isolated from 666 patients with early-stage to late-stage colorectal cancers [80]. A total of 74% of the patients who were positive for the presence of CTCs in blood showed the presence of PD-L1. Furthermore, the presence of PD-L1 on CTCs isolated from breast, prostate, ovary, and gastrointestinal cancers has also been reported as a marker for minimal cellular disease [86,87].

### 5.2. Human Epidermal Growth Factor Receptor 2 (HER2)

Human epidermal growth factor receptor 2 (HER2) is a transmembrane glycoprotein comprising 1255 amino acids that regulate cell proliferation and differentiation [89]. Although no known ligands directly activate HER2, it may inherently be activated or become activated upon heterodimerization with HER3 [90]. Activated HER2 stimulates the proliferation of cells, the formation of new blood vessels, and the migration of cancer cells, thereby playing a critical role in metastasis and release of CTCs into bloodstream. Accordingly, HER2 is overexpressed in 15–30% of invasive breast cancers, with copy numbers varying among cells, exceeding 100 copies per cell in some cases [91,92]. Trastuzumab and pertuzumab are monoclonal antibodies that block HER2 activation and are approved for the treatment of HER2-positive breast cancer patients. Overexpression of HER2 in cancer cells has also been reported in gastric, esophageal, ovarian, endometrial, and lung cancer patients. Therefore, expression of HER2 on the cancer cell surface is an established prognostic and predictive biomarker in cancer therapy.

However, conventional IHC and fluorescence in situ hybridization (FISH) utilize tissue biopsy samples to test the presence of HER2 on cancer cells, which presents technical and logistical challenges [93]. In contrast, the evaluation of HER2 on CTCs enables real-time monitoring in a non-invasive manner. Khandare et al. analyzed HER2 expression on CTCs isolated from 179 breast cancer patients and reported that 64% of patients had CTCs, with 48% of these cells being HER2-positive [94]. Lopes et al. used a microfluidic device, RUBYchip, to capture and enumerate CTCs from metastatic breast cancer patients and compared the efficiency with the FDA-approved CellSearch technology [95]. They also compared the HER2 expression on CTCs at baseline and follow-up with tissue biopsy samples. They observed a 93.3% concordance of HER2 expression on CTCs with the tissue biopsies at the baseline, while the concordance dropped to 40% at follow-up time points. In both cases, RUBYchip performed better than CellSearch. The technology also enabled the detailed characterization of CTC subsets, including those expressing HER2. Incorporating the RUBYchip™ into clinical workflows could address the key limitations of tissue-based testing by enabling the dynamic assessment of HER2 status and supporting more personalized treatment decisions as the disease progresses.

Working with a larger cohort from the DETECT study program, Müller et al. screened 1933 patients with HER2-negative metastatic breast cancer to assess the presence of CTCs and evaluate the correlation between HER2 expression levels and disease outcome [96]. The analysis revealed that patients harboring ≥1 CTC with a strong HER2 signal had shorter overall survival compared to patients with moderate or no HER2 signal, providing strong clinical evidence supporting the use of HER2 expression on CTC as a predictive biomarker

**Figure 5:**
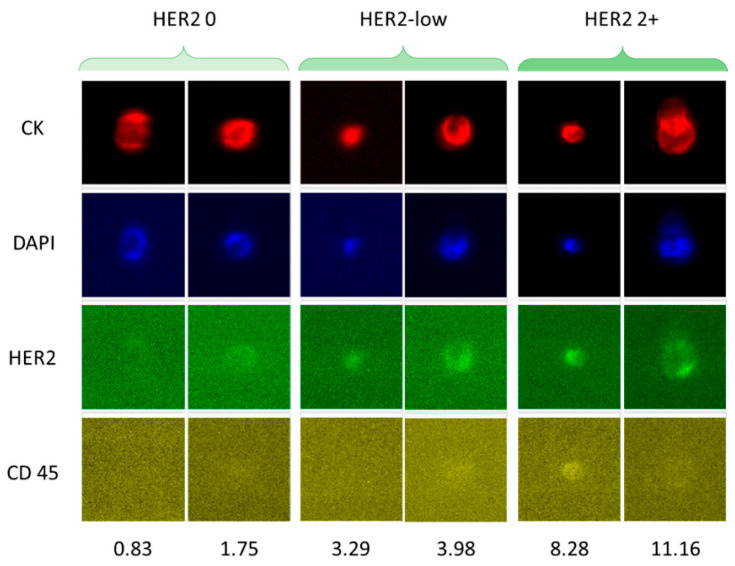
Immunofluorescence-based analysis of CTCs from breast cancer patients with different levels of HER2 expression. The software CellBrowser (https://cells.ucsc.edu/, accessed on 21 January 2026) was used to classify the CTCs as HER2-negative, HER2-low, and HER2 2+. The numbers at the bottom indicate the HER2 signal intensities determined using CellBrowser at 10× magnification. Reprinted from [97] (Open access under the Creative Commons CC BY license; https://creativecommons.org/licenses/by/4.0/, accessed on 21 January 2026).

D’Amico et al. developed a method to select a specific subset of CTCs with low HER2 expression isolated from breast cancer patients using CellSearch technology (Figure 5) [97]. They established a scale from 0 to 3+ for HER2 expression. It was observed that Stage IV breast cancer patients with aggressive disease (defined as ≥5 CTCs per 7.5 mL of blood) experienced poorer outcomes when HER2-low CTCs (1+) were present. Although HER2-positive (3+ value on the proposed scale) CTCs seemed to hold greater predictive relevance for treatment response, the HER2-low population appeared more informative for exploring the biological factors contributing to their prognostic significance. Given the variability in HER2 expression among CTCs, both within a single patient at one time point and across serial samples as the disease evolves, there is a clear need for a standardized diagnostic framework. Thus, incorporating molecular profiling of HER2-low CTCs into clinical workflows will support a real-time assessment of HER2 status and advance the use of CTCs as a distinctive predictive biomarker in metastatic breast cancer.

While the above examples elucidate the prognostic potential of HER2 expression on CTCs, studies highlighting the precision therapeutic potential of HER2 expression on CTCs are also essential. In this context, Meng et al. demonstrated that, amongst 24 breast cancer patients whose original tumors lacked HER2 amplification, 9 showed HER2 gene amplification in their circulating tumor cells as the disease advanced, corresponding to 37.5% of the cohort [98]. Of these nine patients, four received treatment regimens that included trastuzumab. One experienced complete tumor regression, while two achieved partial responses. The authors describe the findings as preliminary, based on a limited patient cohort, and emphasize that larger studies are needed before changing standard treatment. Despite these uncertainties, the results support the continued investigation of CTCs, as they may serve as a minimally invasive, real-time method to monitor the genetic evolution of recurrent cancer.

### 5.3. Epidermal Growth Factor Receptor (EGFR)

Epidermal growth factor receptor (EGFR) is a transmembrane receptor implicated in the pathogenesis and progression of various carcinomas [99]. This has led to an increased interest in developing methods to isolate EGFR-positive CTCs, enumerate them, and monitor the expression profiles throughout treatment. For example, a method to enrich CTCs from NSCLC patients based on EGFR expression has also been reported [100]. Braun et al. reported a positivity rate of 93% for EGFR in 18 high-grade soft tissue sarcoma patients [101]. Whereas Payne et al. studied the expression of EGFR on CTCs isolated from 33 breast cancer patients and observed that the EGFR expression levels remained consistent throughout the treatment period [102].

**Figure 6:**
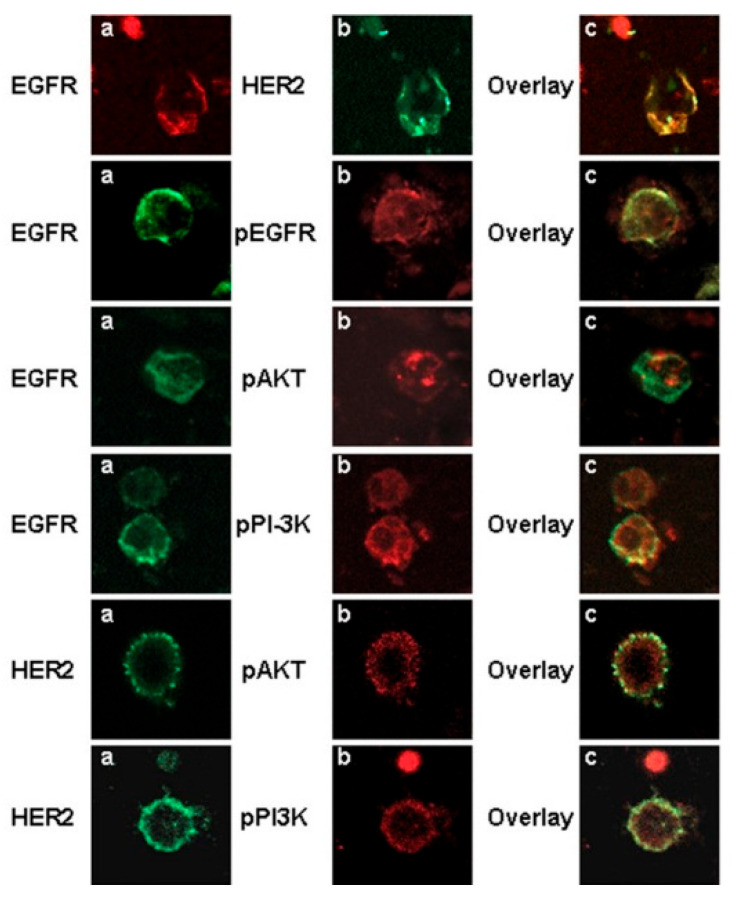
Confocal laser scanning micrographs of CTCs isolated from breast cancer patients using immunomagnetic separation. EGFR was observed to be co-expressed with HER2, pEGFR, phospho-PI3K, and pAkt kinase. (**a**,**b**) are the single channel images of the cells stained with respective antibodies, while (**c**) is the overlay. Magnification 400×. Reprinted from [103] (Open Access under CC BY 2.0; https://creativecommons.org/licenses/by/2.0/, accessed on 21 January 2026).

Kellergi et al. investigated the expression of EGFR and phosphorylated EGFR on CTCs isolated from blood samples of breast cancer patients [103]. They also evaluated HER2 expression and the activation status of the phosphoinositide-3 kinase (PI3K)/Akt pathway in adjuvant and metastatic settings, downstream signaling molecules known to control cell proliferation and migration. They observed that the CTCs co-expressed EGFR, HER2 and phospho-Akt and phospho-PI3K kinases supporting activation of the downstream pathway in metastatic settings, which was not observed in early breast cancer patients (Figure 6). The authors concluded the need to investigate a larger cohort for determining clinical significance. Thus, EGFR profiles of CTCs can aid in selecting eligible patients for anti-EGFR therapy.

### 5.4. Emerging Biomarkers

Trophoblast cell surface antigen 2 (TROP-2) is a promising, underexplored biomarker for the detection of cancers, and was recently shown to be upregulated in triple-negative breast cancer (TNBC) CTCs undergoing epithelial–mesenchymal transition [104]. This is particularly valuable, as unlike traditional epithelial markers such as EpCAM, which are often downregulated during EMT, CTCs retain TROP-2 expression, making it a more reliable marker for detecting cells undergoing transformation [105]. Monitoring TROP-2 expression in CTCs during treatment may also provide predictive insights into the response to TROP-2-targeted antibody–drug conjugates (ADCs), such as sacituzumab govitecan, enabling the real-time assessment of therapeutic response [106]. In addition to TNBC, TROP-2 has also been observed in advanced-stage prostate cancer patients who are resistant to androgen receptor signaling inhibitors, suggesting broader applicability for CTC-based monitoring [107]. Such studies suggest that TROP-2-based therapeutic monitoring of CTCs provides prognostic value; however, further clinical validation is needed to establish TROP-2 as a standard biomarker for CTC-based applications.

Delta-like ligand 3 (DLL3), an inhibitory ligand in the Notch signaling pathway, has minimal expression in adult tissues but is upregulated in various neuroendocrine (NE) malignancies, including small-cell lung cancer (SCLC) and neuroendocrine prostate cancer (NEPC) [108,109,110,111]. Because of its restricted normal tissue expression and strong tumor/neuroendocrine association, it is considered a promising therapeutic target [112]. Recently, multiple groups have reported the utility of the DLL3 expression profile on CTCs in cancer prognosis. Messaritakis et al. studied around 108 treatment naïve SCLC patients and observed that 74.1% had DLL3-positive/CD45-negative CTCs before therapy, with numbers decreasing after the first round of chemotherapy but rising back as the disease progressed [113]. DLL3 was observed to be expressed in CTCs with varying cytokeratin/vimentin status (i.e., epithelial + mesenchymal and mixed phenotypes). Baseline DLL3-positive CTCs were associated with shorter progression-free survival, on the other hand, upon progression, DLL3-positive CTCs were associated with worse overall survival. The advancement of tools to detect on CTCs offers a development of liquid biopsy-based approach to guide DLL3-targeted therapies, such as the ADC SC16LD6.5 (rovalpituzumab tesirine) which has shown promising efficacy in preclinical models of DLL3-positive prostate and lung cancers [114]. However, more prospective studies are needed to validate DLL3-positive CTCs as reliable prognostic and predictive biomarker.

Other emerging biomarkers for predicting outcomes in response to recently developed ADCs include NECTIN-4 and CD70, upregulated in urothelial cancers and renal cell carcinomas respectively [115,116]. However, their expression on CTCs remains largely uncharacterized, highlighting an unmet need and presenting an opportunity to develop dynamic, blood-based assays for non-invasive monitoring and therapeutic stratification.

## 6. Conclusions

The era of targeted therapies demands precision in real-time molecular profiling. The role of blood-based biomarkers, such as CTCs, along with the ctDNA outcome has been well established in a few clinical trials including MIRACLE. ctDNA analysis in establishing MRD has been beneficial in determining the clinical outcome with standard-of-care treatments, in adjuvant settings (DYNAMIC study), in colorectal cancers. While DNA establishes clinical outcome, CTCs, on the other hand, reveal the MRD that could lead to the progression of metastasis due to their capacity to sustain immunological extinction via the presence of the PD-L1 protein. Thus, ctDNA and CTCs complement each other, with CTCs being able to provide cellular context and therapeutic targeting information which ctDNA may not always be able to deliver.

The advent of CTC technologies with protein biomarker expression assays offers roles in patient stratification and selection in early-stage drug discovery, for example, in antibody–drug conjugate discovery programs, when tissue is unavailable, thereby decreasing clinical cycle and expenses. Particularly through surface markers, CTC analysis can enable the clinicians to adapt treatment strategies based on tumor evolution and emerging resistance patterns. As the evidence grows, CTC enumeration and biomarker profiling are poised to augment or even supplant tissue-based approaches in select clinical scenarios.

Moreover, advances in multi-omics, artificial intelligence, single-cell approaches, and organoid modeling are expected to enhance the utilization of biomarkers in disease monitoring. To fully harness their potential in the future, large-scale prospective clinical trials including diverse cancer types will help confirm the predictive value of biomarkers for overall survival, progression-free survival, and treatment response. Additionally, it will be valuable if a global consensus can be achieved regarding quantification of the biomarkers present in each population of CTCs obtained from a single patient. This would further assist oncologists to set up standardized treatment guidelines based on information obtained from molecularly characterized CTCs which would be truly a step towards precision oncology.

## Figures and Tables

**Table 1:** List of different CTC isolation methods and their advantages and disadvantages.

CTC Isolation Method	Isolation Principle	Advantages	Disadvantages	References
Size-based filtration	Separation based on larger size and reduced deformability of CTCs	Label-free method Preserves morphology of CTCs	Small or deformable CTCs may be lost Clogging of filters Size of CTCs may overlap with leukocytes	[60]
Density gradient centrifugation	Separation based on cell density differences	Suitable as a pre-enrichment step	Very low specificity High probability of CTC loss Not suitable as a standalone technique	[52,53]
Microfluidic devices	Separation based on size or antibody interactions with CTCs	Gentle on cells and retains cell morphology Can integrate capture and analysis	Requires the fabrication of complex devices	
Dielectrophoresis	Separation based on capacitance of cell membranes	Label-free identification of CTCs Retains cell viability	Sensitive to buffer conditions	[54]
Immunomagnetic beads-based isolation		Scalable and automatable Reproducible Compatible with clinical workflows	Dependent on presence of markers on CTCs Beads may interfere with imaging and downstream processing	[55,57]

## Data Availability

No new data was created during the writing of this review.
